# Iodine insertion and dispersion of refractive index in organic single crystal semiconductor

**DOI:** 10.1038/s41598-018-21632-2

**Published:** 2018-02-20

**Authors:** Seonho Kwon, Junwan Bae, I. J. Lee

**Affiliations:** Department of Physics, Research Institute of Physics and Chemistry, Chonbuk National University, Jeonju, 54896 Republic of Korea

## Abstract

Insertion of halogens such as bromine or iodine affects the electronic polarizability of ions and the local field inside the medium, and thus modifies the refractive index. Acquiring precise knowledge of the dispersion of refractive index and ultimately tailoring conventional semiconductors for wide-range refractive index control have been a vital issue to resolve before realizing advanced organic optoelectronic devices. In this report, dispersions of the refractive index of a single crystal tetramethyltetraselenafulvalene [C_10_H_12_Se_4_] (TMTSF) are thoroughly studied from broadband interference modulations of photoluminescence (PL) spectra at various temperatures and doping levels. A large enhancement of the refractive index, more than 20% of the intrinsic value, is achieved with inclusion of a small composition of iodide ions, while the structural and optical properties remain mostly intact. Nearly temperature independent dispersion of the refractive index suggests that, unlike most polymers in which the thermal expansion coefficient dominates over the change of polarizability with temperature, the latter enhances significantly and may become more or less comparable to the thermal expansion coefficient given by 1.71 × 10^−4^/K, when single crystal TMTSF is doped by iodine.

## Introduction

Organic crystals of small conjugated molecules, together with conjugated polymers, have attracted much attention because of their fundamental scientific interest and impressive improvements in performance in wide range of organic material based devices, such as organic field effect transistors, organic solar cells, organic light emitting diode displays, organic single crystal lasers^1–4^. As a common base material for the organic electronics, semiconductors of small-molecules offer several advantages over conjugated polymers, which originates from their uniform molecular distribution in nature and consistent synthesis. The organic crystals of small molecules are held together weakly in a solid by van der Waals forces with a well-defined chemical structure. The high degree of order of small molecular crystals allows us to probe fundamental properties without contributions from various extrinsic effects, and thus the systems often play an important role as benchmark systems toward the electronic devices based on thin films. The charge transport, for example, known to limit the performance of the electronic devices, has been one of the leading subjects in which small molecular organic semiconductors have played a major role^1,5–7^. Nevertheless, the fundamental understanding of the charge transport in organic systems, a key to the better performing devices, is still developing area of research. It would require accurate knowledge of both electric and optical parameters from various organic systems with many different structures^1,7^. Among the various optical properties of organic semiconductors, an important but seldom studied issue is that how the electromagnetic wave propagates through the medium depending on wavelength below absorption edge^8,9^. The dispersion of refractive index, essentially linked by absorption spectrum through Kramers-Kronig relation, can provide fundamental information on the energy band structure. Moreover, the effective design and precise fabrication of optoelectronic devices and elements require precise knowledge of the dispersion of refractive index.

Efficient confinement and propagation of light through active control of the refractive index of a medium has been attempted on conventional polymer films in which typical refractive indices are given merely in the range of 1.30–1.70^10^. For better practical applications, the refractive indices of polymers were commonly enhanced in the range of 1.60–1.77 with inclusion of the atoms or organic groups of high molar refraction such as halogens, sulfur atoms, and organometallic moieties^11,12^. However, a maximum enhancement of the refractive index is limited to 1.8, and large substitutions of the doping elements become the cause high optical loss and poor stability. Tailoring conventional organic semiconductors for wide-range refractive index control while maintaining good optical qualities has been remained as a critical issue to resolve before realizing advanced organic optoelectronic devices. Unfortunately, however, the case study for small molecular organic semiconductors is currently very limited^13^. In this report, the dispersions of the refractive index of a small molecular semiconductor TMTSF [C_10_H_12_Se_4_] and their temperature variations are thoroughly studied from the broadband interference modulation of PL spectra in which a pair of the parallel crystal facets takes a role as a cavity resonator of the Farby-Perot type. A large enhancement of the refractive index, more than 20% of the intrinsic value, is achieved with insertion of a small composition of iodide ions, while the structural and optical properties remain mostly intact. The refractive index, depending on its state of polarization and the direction of propagation, has been commonly measured with various techniques such as ellipsometry, interferometry, and prism or grating coupling methods. Our study strongly suggests that analysis of the interference modulation of the PL spectra is a simple but powerful tool for monitoring the dispersions and their temperature dependence of organic single crystal semiconductors in which the applications of these common techniques are not always straightforward.

## Data and Discussions

**Figure 1:**
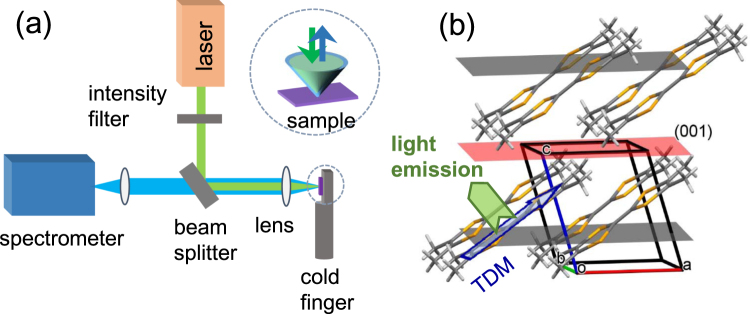
(**a**) Photoluminescence setup of the single crystal TMTSF. The laser excitation and PL detection are performed on a small spot on the (001) facet. (**b**) Structure of single crystal TMTSF in which the triclinic unit sell contains single TMTSF molecule.

**Figure 2:**
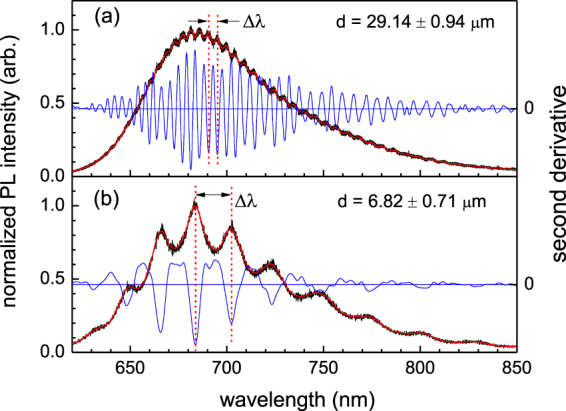
Representative PL spectra taken from two different samples. PL spectrums of the thick (d = 29.14 ± 0.94 μm) and thin samples (d = 6.82 ± 0.71 μm) are measured at 270 and 298 K, respectively. Smoothed curve obtained by adjacent averaging of the normalized raw data and its second derivative are shown in thin solid lines.

**Figure 3:**
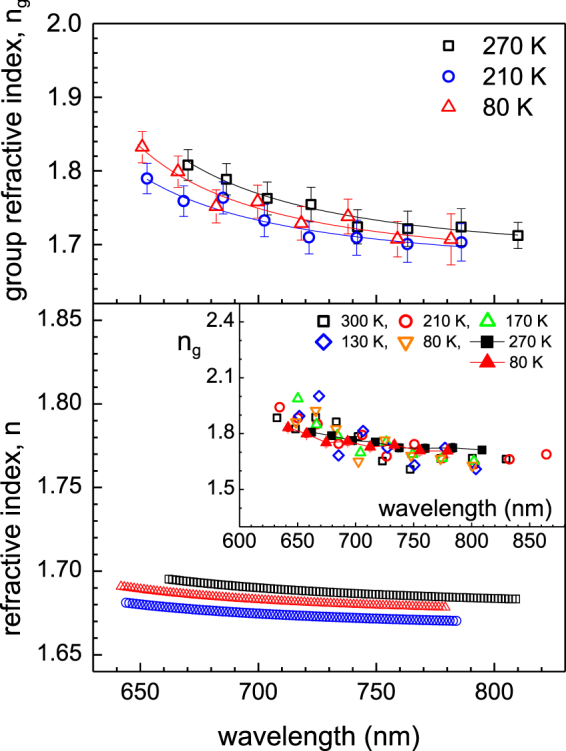
The dispersions of group (upper panel) and phase (lower panel) refractive index at various temperatures. Inset shows the dispersions determined from both thin (d = 6.82 ± 0.71 μm) and thick (d = 29.14 ± 0.94 μm) samples. Data displayed with line-and-filled symbols in inset are taken from the thick sample at 270 and 80 K.

The experimental setup for PL measurement is shown in Fig. 1(a). The excitation light was aligned normal to the (001)-plane of the single crystal TMTSF and the emission spectra was detected along the same direction (c*-axis). Figure 1(b) constructed based on single crystal x-ray diffraction measurements displays that the crystal structure is a triclinic with space group P-1. Unlike most studied organic semiconductors such as pentacene and rubrene which exhibit a herringbone packing motif^1^, the planar TMTSF molecules forms a slipped face-to-face arrangement along the a-axis with a pitch angle of ~39° and packs in a brick-wall pattern within the (010) plane providing an efficient two-dimensional network of charge transport. A possible orientation of transition dipole moment (TDM) which likely coincides with the long molecular axes is indicated with an arrow. Representative PL spectra taken from samples of different thicknesses, d = 29.14 ± 0.94 μm and d = 6.82 ± 0.71 μm, are displayed in Fig. 2(a,b), respectively. The data plotted on the right axis display the second derivative of the traces obtained from adjacent average of the normalized raw data. A fair level of objectiveness in determining the modulation peak positions is maintained by taking the dips of the second derivative. The frequency of the intensity modulations which overlay a monotonic emission spectrum for the broad range of wavelength is highly dependent on the sample thickness. Data also indicate that there exist considerable surface emissions in the TMTSF system, which is somewhat unusual considering the fact that the surface emissions were negligible in similar measurements on common organic single crystals such as rubrene and thiophene/phenylene co-oligomers^4,8,9^. The propagation of the molecular light emission is associated with the orientation of the molecular transition dipole moments (TDM)^14,15^. Contrary to the common organic single crystals exhibiting nearly upright configuration of the TDM to the layered *ab*-plane, the planar TMTSF molecules intersect the (001)-plane at ~39°. The intersecting angle seems sufficiently large enough to allow the majority of the molecular light emissions to leak out through the (001)-facets resulting in the surface emission as observed. Moreover, it is expected that the intersecting angle being close to the critical angle for the total internal reflection would make intensity of the surface emission extremely sensitive to the modification of refractive index. The wavelength spacing between adjacent modulation peaks (Δλ), the so-called free spectral range, is inversely proportional to the thickness of the single crystal or, more precisely, the separation between two end-facets along the detecting direction of the light emission. In fact, a pair of parallel facets functions as a Fabry-Perot type resonator within which multiple reflections generate interferences resulting in the intensity modulation^4,8,9,14^. Only standing waves at discrete wavelength can exist in the self-cavity resonator. The condition for the constructive interference for the self-cavity of length *L*, which is essentially the longitudinal cavity multimode confined as standing waves, is given by

1 $$N=\frac{2nL}{\lambda }=2nL\nu ,$$

where *ν* (=1/*λ*) is the wavenumber in vacuum, *n* is the phase refractive index, and *N* is the longitudinal mode order (integer). Thus, the longitudinal mode spacing is obtained as

2 $$\delta N=2n(1+\frac{\nu }{n}\frac{\delta n}{\delta \nu })L\delta \nu =2{n}_{g}L\delta \nu ,$$

where *n*_*g*_ is the group refractive index associated with the speed of electromagnetic wave packet. The wavelength dependent group refractive indices are determined using the measured *δN*, *δν*, and the cavity length or sample thickness *L*. For the thick sample (*L* = 29.14 ± 0.94 μm) with high frequency modulations, the mode order spacing of *δN* = 4 which covers δν in the range between 370 and 405 cm^−1^ depending on the wavelength and temperature is used to determine the group refractive indices. The mode order of *δN* = 1 is employed for the thin sample (*L* = 6.82 ± 0.71 μm) with slow modulations. Based on the self-cavity resonator model described by Eq. (2), representative dispersions displayed in inset of Fig. 3 are constructed from both thin and thick samples at various temperatures (The linear thermal expansion coefficient (LTEC) along the relevant (001)-facet was estimated based on our single crystal XRD data taken at 200 and 298 K. The interlayer spacing *L*(001) = *V*/(*ab* sin γ) is obtained as 7.256 and 7.238 Å at 298 and 200 K, respectively. Thus the LTEC per K (i.e., (1/*L*) *dL*/*dT*) is given as 2.537 × 10^−5^/K from which all the sample thickness above 80 K are estimated using the known thickness at 298 K. The volume expansion coefficient is determined as *β* = 1.706 × 10^−4^/K based on lattice parameters). The dispersions determined from the thin sample with slower intensity modulations are more scattered than the data obtained from the thick sample, which is because the uncertainty involved in the estimate of the indices is associated with the modulation-width or the quality factor of the cavity resonator (see Fig. 2). Nevertheless, we notice that the dispersions of refractive index are obtained consistently for both samples independent of the sample thickness, which justifies the data analysis. Data from the thick sample with a better resolution are displayed in the main panel in which an average uncertainty ±0.023 is given in the estimate of the refractive indices. It is not clear at the moment whether the dispersions of the refractive index shift downward with increasing temperature, as expected for most polymers. We will discuss this issue in more detail later.

The dispersion of light in medium is empirically described by Sellmeier formula. The phase refractive index is often calculated utilizing the first order Sellmeier equation^16,17^ given by

3 $$n=\sqrt{A+\frac{B}{1-{(C/\lambda )}^{2}}},$$

where the fitting parameter *A* and *B* are dimensionless and the parameter *C* has a length scale associated with resonant absorption wavelength. At long wavelengths far from the absorption peak, the phase refractive index is approximately given by $n \sim \sqrt{A+B}$. With the substitution of the Sellmeier formula for the phase refractive index in Eq. (2), the dispersion of the group refractive index in a function of the three fitting parameters is obtained^8,9^. The thin lines through the data points shown in upper panel of Fig. 3 are the outcomes of the least square fitting of the corresponding set of data points. Finally, the dispersion of the phase refractive index is calculated with application of the three fitting parameters to the Sellmeier equation Eq. (3), which is displayed in lower panel of Fig. 3. The phase refractive index is given as a monotonically decreasing function between *n* = 1.70 and 1.68 in the wavelength range of 640–810 nm. The refractive index at the sodium D-line (589.3 nm) and at the long wavelength limit are estimated by *n*_D_ = 1.73 ± 0.023 and *n*(λ→∞) = 1.66 ± 0.01 at 270 K, respectively. The resonant absorption wavelength or the parameter *C*, which is closely related to the energy band gap, was obtain as 537 ± 40, 526 ± 60, and 542 ± 44 nm at 270, 210, and 80 K, respectively. The nominal value of *C* ≈ 535 nm corresponding energy gap of an order of 2.3 eV is close to that of the single crystal rubrene^8^, one of the most intensively studied organic materials.

**Figure 4:**
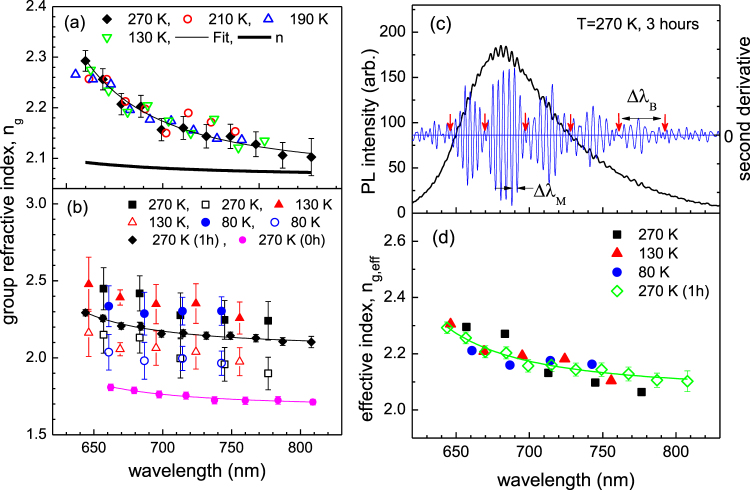
The dispersions of group refractive index determined at various temperatures. Panel (a) shows the data taken after the sample was doped for 1 hour. Data with thick line indicates an estimate of the corresponding phase refractive index *n*. Panel (b) displays the dispersions of the 3 hour-exposed at 270, 130, and 80 K, in which a two-resonator-model is assumed. The dispersions taken at 270 K for the intrinsic sample (0 h) and the 1 hour-exposed (1 h) are also plotted with fitting lines for comparison. Panel (c) displays a representative PL spectrum and its second derivative taken at 270 K after 3 hours exposure of iodine vapor. The modulation wavelength (Δλ_M_) and beating wavelength (Δλ_B_) are indicated. Panel (d) shows the effective group refractive indices, in which the mixing of two resonators with nearly equal volume fraction is assumed.

Panel (a) and (b) of Fig. 4 display the dispersions of group refractive index taken at various temperatures after the samples are exposed to iodine vapor for 1 hour (panel (a)) and for 3 hours (panel (b)). Data displayed in panel (a) show that all the temperature variations of the group refractive indices are well described by the dispersion of the 1 hour-exposed taken at 270 K within a range of error ±0.034 at most. The resonant absorption wavelength is determined as C = 540 ± 20 nm and the phase refractive indices obtained at the sodium D-line and at the long wavelength limit are *n*_D_ = 2.13 ± 0.024 and *n*(λ→∞) = 2.06 ± 0.024, respectively. Interestingly, a quasi-periodic beating pattern is observed when the sample is exposed for 3 hours as shown in panel (c). Both the envelope (beating) and modulation wavenumber slightly decrease as the wavelength increase. The clear beating behavior which consistently appears at different temperatures seems to suggest that there exist two different sets of broadband interference modes with slightly different free spectral ranges. The modulation wavenumber is given by 1/Δ*λ*_M_ = (*ν*_1_ + *ν*_2_)/2 and the beating wavenumber is obtained as $1/{\rm{\Delta }}{\lambda }_{B}=|{\nu }_{1}-{\nu }_{2}|$, in which *ν*_1_ and *ν*_2_ are defined by inverse of the free spectral range for corresponding each resonator modes. Then, the values *ν*_1_ and *ν*_2_ are given by the combination of the modulation and beating wavenumbers, that is 1/Δ*λ*_M_ ± 1/(2Δ*λ*_B_) in which the (+) sign is designated to the resonator 1 and the (−) sign is assigned to the resonator 2, respectively. In terms of the free spectral range, the group refractive index in Eq. (2) can be re-expressed by

4 $${n}_{g}=\frac{{\lambda }^{2}}{2L{\rm{\Delta }}\lambda },$$

where the mode order spacing is set to *δN* = 1. The modulation wavelength (Δ*λ*_M_) is averaged within each envelope of the beating patterns and *λ* is taken as the central wavelength of the beating envelope. In the panel (b) of Fig. 4, the scattered data points with the filled and open symbols at various temperatures are the results obtained for the 3 hour-exposed. Data taken at 270 K from the intrinsic (0 h) and the doped for 1 hour (1 h) are also displayed with filled circles, diamonds, and associated fitting lines for comparison. Nearly independent of the temperature variations, the central value of the 3-hour data either shifts up (resonator 1) or down (resonator 2) by a constant value (~0.2) from the dispersion of the 1-hour. The splitting of the refractive index due to the presence of two closely related dispersions likely implies a presence of non-uniform distributions of the dopant. The buildup of non-uniform distribution of iodine concentration has been also observed in pentacene films when the films were doped for extended duration^18^. In the absence of the resonator 2, the system would follow the dispersion of the resonator 1 and vice versa. For randomly arranged two kinds of resonators, the effective group refractive index can be calculated using the Bruggeman mixing formula given by

5 $${f}_{1}(\frac{{n}_{1}^{2}-{n}_{g,eff}^{2}}{{n}_{1}^{2}+2{n}_{g,eff}^{2}})+(1-{f}_{1})(\frac{{n}_{2}^{2}-{n}_{g,eff}^{2}}{{n}_{2}^{2}+2{n}_{g,eff}^{2}})=0,$$

where *f*_1_ is the volume fraction of the resonator 1. As an example, we show in Fig. 4(d) that, with a proper setting of the volume fraction in the range between 0.46 and 0.59, the dispersion of the effective index becomes coincident with that of the 1-hour data. When sample is further exposed by iodine vapor for 10 hours, in comparison with the 1-hour data, the beating wavenumber is enhanced, suggesting larger shifts in the dispersions. Indeed, one resonator with a higher set of indices closely follows the trace of the 1-hour data (1 h) and the other intimately matches the trace of the intrinsic data (0 h). The effective refractive indices become reduced as the duration of doping is increased from 3 to 10 hours. In other words, the optimum doping occurs with inclusion of a small composition of iodide ions in the range of 0.007.

**Figure 5:**
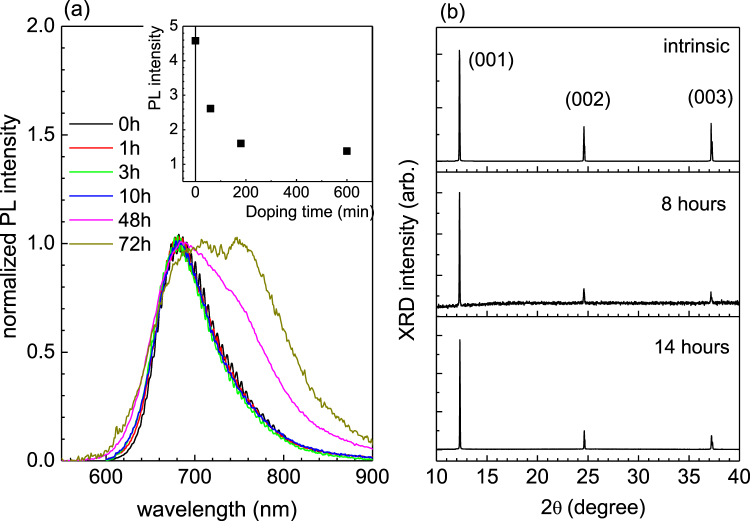
Panel (a) displays normalized PL lineshapes at T = 270 K for various doping durations. Inset shows integrated PL intensity in arbitrary unit taken at T = 270 K. Panel (b) exhibits XRD (2θ scan) curves of single crystal TMTSF treated for different doping durations, the intrinsic, 8 hours, and 14 hours at room temperature.

The optical excitation of an organic semiconductor leads to the formation of excitons i.e., the tightly bound molecular excited states residing between the highest occupied molecular orbital (HOMO) and the lowest unoccupied molecular orbital (LUMO) levels. Doping of iodine introduces impurities and disorders, and provides additional nonradiative decay channels to diffusing excitons. Indeed, inset of Fig. 5(a) exhibits that the PL intensity decreases sharply upon the introduction of iodine vapor to the TMTSF single crystal. With 3 hours exposure to iodine vapor which corresponds to a small composition of iodide ions in the range of ~0.007, the integrated PL intensity decreases abruptly to a saturation, near 30% of the initial value. However, contrary to the acute sensitivity of the PL intensity to the iodine exposure, the PL linewidth and its shape remain more or less the same even with 10 hours of doping which gives the iodine composition in the range of 0.043. The interference modulations fade away as the duration of the exposure extends more than 10 hours. Taking a closer look at the PL spectra below the 10-hour trace, we notice that the spectra shift to higher energy side by ~11 meV with the doping. The small blueshift of the spectra can be attributed to the reduction of exciton binding energy with the increase of carrier density. A similar case was found in a 2D transition-metal dichalcogenide semiconductor in which doping was controlled by gate voltage in a configuration of the field effect transistor^19^. Unlike the electrical doping by gate voltage, the chemical doping inevitably induces disorders and even causes structural damage when doped excessively. When iodine vapor is introduced to organic crystal, it is known that iodide ions ${{\rm{I}}}_{3}^{-}$ and ${{\rm{I}}}_{5}^{-}$ are formed as a result of interactions with host molecules^18,20,21^. The iodide ions diffuse into interstitial sites in-between and alongside the host molecules^20,21^. The panel (b) of Fig. 5 showing x-ray diffraction experiments indicates that there occurs no noticeable change in the crystal structure accompanied by the introduction of iodine vapor with up to 14 hours of exposure. Data suggest that the iodide ions likely sit in the interstitial sites between the face of TMTSF molecules in (010)-plane under strong interaction with the Se atoms. When the specimen is doped for 48 hours, the spectrum linewidth increases due to the enhanced vibronic progression (sideband) of the main 680 nm band, or due to relatively stronger suppression of the main band. The resulting decrease in the peak ratio, i.e. the main 680 nm band over the progression, is consistent with a strong reduction of the exciton coherence length by disorders including structural defects^22,23^. Alternatively, we note that a similar suppression of the peak ratio can be described by a J-type aggregate in which the intensity of the main band decreases markedly with increasing disorders^24^. In fact, the molecular packing in the form of a slipped face-to-face with pitch angles less than 54° is consistent with the J-aggregates. When the samples are exposed by iodine vapor for less than 10 hours, the amount of induced disorder is sufficiently low and not enough to modify the lineshapes of the PL spectra. The case should be comparable to the carrier doping by gate voltage^19^, in which the blueshift of exciton energy as well as the gradual decrease of PL intensity are attributed to the increasing carrier density. Thus, it is expected that the PL intensity gradually decreases with doping duration within the low doping regime below 10 hours. On the contrary, as displayed in inset of Fig. 5(a), an acute drop in the PL intensity even with a small inclusion of iodide ion is observed, which cannot be explained by the increased carrier density alone. It must be associated with the enhancement of the refractive index which would effectively prevent the emissions from leaking out through the surface due to smaller critical angle for the total internal reflection. The abrupt decrease in the PL intensity in the early doing regime is a manifestation of the efficient confinement of light through active control of the refractive index of medium.

The refractive index, in relation with the density and polarizability, is described by the Lorentz-Lorentz equation:

6 $$\frac{{n}^{2}-1}{{n}^{2}+2}=\frac{N\rho \alpha }{3M{\varepsilon }_{0}},$$

in which *N* is Avogadro’s number, *ρ* is density, *α* is polarizability, *M* is molecular weight, and *ε*_0_ is free space permittivity. The refractive index can be effectively increased by introduction of substituents with high molar refraction *R* which is related to the polarizability *α* of the molecules constituting the medium by $R=N\alpha /3{\varepsilon }_{0}$. Halogen elements, such as iodine and bromine, were utilized for developing high refractive index polymers in 1992^11^. The temperature dependence of refractive index (*dn/dT*), so-called thermo-optic coefficient, is determined by the combined contributions from the polarizability and thermal expansion coefficient. By differentiating the Lorentz-Lorentz equation Eq. (6), one obtains the following equation^25^:

7 $$\frac{dn}{dT}=\frac{({n}^{2}-1)({n}^{2}+2)}{6n}(\frac{1}{\alpha }\frac{d\alpha }{dT}-\frac{1}{V}\frac{dV}{dT}).$$

The thermo-optic coefficient is positive when the change of polarizability with temperature (polarizability coefficient) is larger than that of the thermal expansion coefficient. For common polymers, the thermal expansion coefficient dominates over the polarizability coefficient and the latter can be neglected^26^, which leads to a negative refractive index change with increasing temperature. For the intrinsic sample with the phase refractive index at the D-line *n*_D_ = 1.70 ± 0.023 at T = 210 K and the volume thermal expansion coefficient *β* = 1.71 × 10^−4^/K, thermo-optic coefficient is determined by −4.47 × 10^−4^/K which places the TMTSF system in the high-end category among common polymers. The expected change in the refractive index is determined by Δ*n* = 0.058 when temperature decreases to T = 80 K. When temperature increases to T = 270 K, the change of the refractive index is obtained as Δ*n* = −0.027. The expected changes in the refractive index with the temperature variations are either an opposite direction or falling a bit short of the experimental data displayed in lower panel of Fig. 3. Nevertheless, it is important to account that the expected changes are comparable, well within the range of experimental uncertainty ±0.023. For the intrinsic sample, the expected changes of the refractive index with the temperature variations may have been not large enough to be identified above the experimental uncertainty. For the 1hour-doped sample, if we take the usual assumption for polymers that the polarizability coefficient is negligible in comparison to the volume expansion, the thermo-optic coefficient drastically increases to −1.40 × 10^−3^/K. The corresponding changes in the refractive index increase by threefold in comparison with the intrinsic case, i.e. Δ*n* = 0.174 (from 210 to 80 K) and Δ*n* = −0.081 (from 210 to 270 K) which should be large enough to be recognized above the experimental uncertainty. However, as displayed in Fig. 4(a) the dispersions are given nearly temperature independent, which imply that contrary to the common assumption the polarizability coefficient is enhanced dramatically and may even become more or less comparable to the volume expansion coefficient 1.71 × 10^4^ /K, when the single crystal TMTSF is doped by iodine for 1 hour.

So far there has been only a few studies on the TMTSF system, all of which focus on electrical transport^27–29^. The latest study suggests that the system has a good electrical mobility in the range of μ ~ 4 cm^2^/V at room temperatrue^29^. With the added means to tune and monitor the optical characteristics that we have shown, we expect that the TMTSF single crystal should serve as a new benchmark system succeeding pentacene and rubrene semiconductors, both of which have provided significant insights into the fundamental understanding of organic semiconductors.

## Conclusions

The dispersion of the refractive index and its temperature dependences of organic single crystal tetramethyltetraselenafulvalene (TMTSF) are systematically studied from the broadband interference modulation of photoluminescence (PL) spectra, which is essentially the emission spectra coupled to the longitudinal cavity multimode formed naturally between a pair of the parallel crystal facets. The dispersions of refractive index monotonically decrease in wavelengths between 640–810 nm, which are well described by the first order Sellmeier dispersion formula. With an exposure of iodine vapor for 1 hour which gives fractional compositions much less than 0.007, the refractive index of the singel crystal TMTSF is largely enhanced more than 20%, while the crystal maintains its structural and optical integrity. Non-uniform distribution of the dopant is manifested by the beating patterns in the interference modulations of the emission spectra when the system is exposed for extended durations. The effective dispersion of refractive index is reduced as the composition of iodine increases to 0.043 ± 0.012 with 10 hours of the exposure. It is demonstrated that the analysis of the interference modulation of PL spectra is a simple but powerful tool for understanding the dispersion of refractive index of organic single crystals at various temperatures and doping levels. The precise knowledge on various optical characteristics would be a vital prerequisite for design and fabrication of advanced optoelectronic devices.

## Methods

As purchased powder form of TMTSF (>97%, Sigma-Aldrich) was purified by multiple sublimation and solidification under high vacuum. The purified powder was sublimated at 160 °C and carried by a continuous flow of Argon gas (ultrahigh purity Ar gas at a flow rate 80 cc/min) in quartz tube at ambient pressure. Single crystal TMTSFs were grown downstream in the gas flow at the range of temperature between 140 and 130 °C. The crystal structure was analyzed based on data taken at two temperatures of 298 and 200 K. Bruker SMART APEX CCD diffractometer was equipped with a graphite monochromator, which provided Mo K_α_ line of λ = 0.7107 Å. Doping of TMTSF was performed in a upside-down U-shaped glass chamber. One end of the glass chamber containing iodine source (≥99.99%, Sigma-Aldrich) was cooled to 241 K, which essentially determined the iodine vapor pressure of ~1 mTorr that would be exposed to single crystal TMTSF. The other side of the diffusion chamber consisting of several doping cells was pumped below 1 mTorr before the iodine vapor was introduced to diffuse through the samples residing in each cell at room temperature. Each doping cell equipped with an independent valve allows us to stop the doping process on a specific cell, while we continue doping on other cells. All samples were pre-cleaned with isopropyl alcohol of HPLC grade and annealed at 60 °C for 2 hours before they were loaded to each cell. A single crystal of a uniform thickness was cleaved to several pieces of submillimeter-square in sizes and each piece was treated with different doping conditions, which essentially allowed us to isolate the doping effect from the data taken under various experimental conditions. The chemical compositions of iodine for given various durations of the exposure were determined by an electron probe micro-analyzer (EPMA-1600). With 5 and 10 hours of doping, the compositions of iodine were measured as 0.018 ± 0.006 and 0.043 ± 0.012, respectively. Measurements taken on 4 samples between 5 to 13 hours of exposure indicate that the composition of iodine depends linearly on the doping duration followed by ~90 minutes of lead time between openings of the doping cell and starting of the effective doping. Based on the doping pattern with the exposure time, a composition of ~0.007 is expected when the sample is exposed for 3 hours. The iodine composition was under the detection limit when sample was doped for 1 hour. We note from the measurements that the weakly bonded nature of the organic molecular single crystal inherently limits the detection of EPMA in the range of ~0.01. A micro PL/Raman measurement system, LabRAM HR UV/Vis/NIR spectrometer (by HORIBA Jobin Yvon) with a continuous 514 nm (2.41 eV) excitation line, was used for the PL measurements. The size of laser spot, approximately diameter of 1 μm, was aligned to one of samples mounted on a cold-stage which was capable of controlling a stable temperature between 10 and 298 K. The power of the laser excitation was varied from 0.05 to 2.5 mW at 14 K and no noticeable change was found in the PL lineshape. A typical power level of 1.5 mW was maintained throughout the PL measurements as long as it warranted a reasonable signal to noise ratio. In each measurement spot, multiple traces were obtained and carefully examined for possible degradation and/or spontaneous heating effect by the focused laser beam.

### Data availability

The datasets generated during and/or analysed during the current study are available from the corresponding author on reasonable request.

## References

[1] Dong H, Fu X, Liu, Wang JZ, Hu W (2013). 25th Anniversary Article: Key Points for High-Mobility Organic Field-Effect Transistors. Adv. Mater..

[2] Mishra A, Bauerle P (2012). Small Molecule Organic Semiconductors on the Move: Promises for Future Solar Energy Technology. Angew. Chem. Int. Ed..

[3] Reineke S (2009). White organic light-emitting diodes with fluorescence tube efficiency. Nature.

[4] Ichikawa M (2005). Laser oscillation in Monolithic (undoped) molecular single crystals. Adv. Mater..

[5] Podzorov V (2004). Intrinsic Charge Transport on the Surface of Organic Semiconductors. Phys. Rev. Lett..

[6] Jurchescu OD, Popinciuc M, van Wees BJ, Palstra TTM (2007). Interface-Controlled, High-Mobility Organic Transistors. Adv. Mater..

[7] Sirringhaus H, Sakanoue T, Chang J-F (2012). Charge-transport physics of high-mobility molecular semiconductors. Phys. Status Solid B.

[8] Sugimoto S, Fukunishi Y, Fukaya Y, Yamao T, Hotta S (2013). Refractive Index Dispersion and Anisotropic Group Refractive Indices of RubreneCrystals, *Trans*. Mat. Res. Soc. Japan.

[9] Yamao T, Okuda Y, Makino Y, Hotta S (2011). Dispersion of the refractive indices of thiophene/phenylene co-oligomer single crystals. Journal of applied physics.

[10] Brandrup, J., Immergut, E. H., Gruike, E. A., Abe, A. & Bloch, D. R. *Polymer Handbook, 4th ed*. (John Wiley & Sons, New York, 2005).

[11] Minns RA, Gaudiana RA (1992). Design and synthesis of high refractive index polymers. II, J. M. S. Pure Appl. Chem..

[12] Liu J-G, Ueda M (2009). High refractive index polymers: fundamental research and practical applications. J. Mater. Chem..

[13] Yokoyama D, Nakayama K, Otani T, Kido J (2012). Wide-Range Refractive Index Control of Organic Semiconductor Films Toward Advanced Optical Design of Organic Optoelectronic Devices. Adv. Mater..

[14] Bisri SZ (2009). High Mobility and Luminescent Efficiency in organic single-crystal light-emitting transistors. Adv. Funct. Mater..

[15] Bando K (2008). Optical selection rule for the lower Davydov excitons in co-oligomer single crystals. Phys Rev B.

[16] Gorachand. G (1997). Sellmeier Coefficients and Dispersion of Thermo-Optic coefficients for some optical glasses. Applied Optics.

[17] Peng HJ (2002). Optical constants of methyl-pentaphenylsilole by spectroscopic ellispometery. J. Appl. Phys..

[18] Jakavovic J (2011). Surface and interface analysis of iodine-doped pentacene structures of OTFTs. Surf. Interface Anal..

[19] Shang J (2015). Observation of Excitonic Fine Structure in a 2D Transition-Metal Dichalcogenide Semiconductor. ACS Nano.

[20] Cazayous M (2004). Iodine insertion in pentacene thin films investigated by infrared and Raman spectroscopy. Phys. Rev. B.

[21] Minakata T, Nagoya I, Ozaki M (1991). Highly ordered and conducting thin film of pentacene doped with iodine vapor. J. Appl. Phys..

[22] Ahn T-S (2008). Experimental and theoretical study of temperature dependent exciton delocalization and relaxation in anthracene thin films. J. Chem. Phys..

[23] Spano FC (2002). Absorption and emission in oligo-phenylene vinylene nanoaggregates: The role of disorder and structural defects. J. Chem. Phys..

[24] Spano FC (2010). The spectral signatures of frenkel polarons in H- and J-aggreates. Acc. Chem. Res..

[25] Tan CZ (1998). Review and analysis of refractive index temperature dependence in amorphous SiO_2_. J. Non-Cryst. Solids.

[26] Zhang Z, Zhao P, Lin P, Sun F (2006). Thermo-optic coefficients of polymers for optical waveguide applications. Polymer.

[27] Nam MS, Ardavan A, Cava RJ, Chaikin PM (2003). Intrinsic electronic transport properties of organic field-effect transistors based on single crystalline tetramethyltetraselenafulvalene. Appl. Phys. Lett..

[28] Kim JY, Yun MY, Jeong D-W, Kim J-J, Lee IJ (2009). Structural and electrical properties of the single-crystal organic semiconductor tetramethyltetraselenafulvalene (TMTSF). J. Korean Phys. Soc..

[29] Xie H, Alves H, Morpurgo AF (2009). Qualitative analysis of density-dependent transport in tetramethyltetraselenafulvalene single-crystal transistors: Intrinsic properties and trapping. Phys. Rev. B.

